# Erratum: Livashvili et al. Appearance of a Solitary Wave Particle Concentration in Nanofluids under a Light Field. *Nanomaterials* 2021, *11*, 1291

**DOI:** 10.3390/nano11082084

**Published:** 2021-08-17

**Authors:** Abram I. Livashvili, Victor V. Krishtop, Polina V. Vinogradova, Yuriy M. Karpets, Vyacheslav G. Efremenko, Alexander V. Syuy, Evgenii N. Kuzmichev, Pavel V. Igumnov

**Affiliations:** 1 Institute of Natural Sciences, Far Eastern State Transport University, 47, Seryshev St., 680021 Khabarovsk, Russia; livbru@mail.ru (A.I.L.); vpolina17@hotmail.com (P.V.V.); kjum1947@mail.ru (Y.M.K.); oblako3@yandex.ru (V.G.E.); 2 Department of General Physics, Perm National Research Polytechnic University, 29, Komsomolsky Prospekt, 614990 Perm, Russia; 3 Department of General Physics, Moscow Institute of Physics and Technology, 9, Institutskiy Per., 141701 Dolgoprudny, Russia; alsyuy271@gmail.com; 4 Institute of Materials Technology of Khabarovsk Centre of FEC The Russian Academy of Sciences, 153, Tihookeanskaya St., 680042 Khabarovsk, Russia; e_kuzmichev@mail.ru (E.N.K.); 407320@mail.ru (P.V.I.)

The authors wish to make the following corrections to this paper [1]. The *div* was omitted due to misprints which appeared in revised version.

The content from Equations (1) and (2) in Section 2 needs to be corrected. The updated information is included below:

(1) $$C_{p}\rho \frac{\partial T}{\partial t}=div\left(\lambda \left(C\right)\;\left(\vec{gradT}\right)\right)+\alpha \left(C\right)I_{0},$$

(2) $$\frac{\partial C}{\partial t}=div\left(D\vec{gradC}\right)+D_{T}div\left(C\left(1-C\right)\vec{gradT}\right)-\vec{V\cdot }\vec{gradC},$$

The cited reference [27] needs to be corrected. The updated information is included below:

It should be noted that, in Equation (2), we take into account the incompressibility of the nanofluid: *div* $\vec{V}$ = 0 [27].

The content from Equations (3) and (4) in Section 2 needs to be corrected. The updated information is included below:

(3) $$div\left(\lambda \left(C\right)\frac{\partial T}{\partial x}\right)\approx \lambda \left(C\right)\frac{\partial^{2}T}{\partial x^{2}},\;div\left(D\frac{\partial C}{\partial x}\right)\approx D\frac{\partial C}{\partial x},$$

(4) $$div\left(C\left(1-C\right)\vec{gradT}\right)\approx C\left(1-C\right)\frac{\partial^{2}T}{\partial x^{2}},$$

The References section needs to be corrected. The book title in Ref. [26] was incorrect. The updated information is included below:

26. Landau, L.D.; Lifshitz, E.M. *Fluid Mechanics*, 2nd ed.; Pergamon: London, UK, 1987.

The changes do not affect the scientific results or conclusions in the original published paper.

The authors would like to apologize for any inconvenience caused to the readers by these changes.
